# Assessment of Carotid Arterial Wall Elasticity in Type 2 Diabetes Mellitus Patients with Microalbuminuria by Real-Time Ultrasound Elastography

**DOI:** 10.1155/2012/340974

**Published:** 2012-05-30

**Authors:** Yu-Hong Zhang, Yuan Gao, Ben-Li Su

**Affiliations:** ^1^Department of Diagnostic Ultrasound, Second Affiliated Hospital of Dalian Medical University, Dalian 116023, China; ^2^Department of Endocrinology, Second Affiliated Hospital of Dalian Medical University, Dalian 116023, China

## Abstract

The aim of this study was to evaluate carotid arterial wall elasticity in type 2 diabetes mellitus (T2DM) with microalbuminuria by real-time ultrasound elastography. Two hundred and ten T2DM patients were divided into two groups according to levels of urinary albumin excretion (UAE): T2DM without microalbuminuria (T2DM1 group, 120) and T2DM with microalbuminuria (T2DM2 group, 90). The right common carotid arteries were examined by real-time ultrasound elastography. The strain ratio (SR, blood to arterial wall strain ratio) was calculated by dividing the strain value of the blood by that of the carotid arterial wall. The correlation between SR and general data was analyzed. The mean SR value ± SD of T2DM2 group was significantly higher than that of T2DM1 group (*P* < 0.05). SR was positively and significantly correlated with UAE, HbA1c, and systolic blood pressure (*r* = 0.456,0.435,0.235, *P* < 0.05 for all). The mean value ± SD of UAE, HbA1c, 2hPG, BMI, and TC of T2DM2 group was significantly higher than that of T2DM1 group (*P* < 0.05 for all). In conclusion, there is an association between microalbuminuria and carotid arterial wall elasticity in T2DM patients.

## 1. Introduction

The major complication of type 2 diabetes mellitus (T2DM) is atherosclerosis, and it is the main cause of death in diabetic patients [1–3]. However, the mechanism remains poorly understood, especially the direct relation between microalbuminuria and vascular wall elasticity in T2DM. Several researches studied the relation between arterial stiffness and microalbuminuria in diabetes by pulse wave velocity (PWV) and echo-tracking technique [4–6]. In this study, we used real-time ultrasound (US) elastography to evaluate the elasticity of carotid arterial wall in T2DM with microalbuminuria. The real-time US elastography is a newly developed ultrasound technique which provides an estimation of tissue stiffness. And it became a focus in recent years. The US elastography has been mainly applied to the studies of breast, thyroid gland, and prostate [7–9].

## 2. Materials and Methods

### 2.1. Participants

The study included 210 consecutive patients of T2DM in the Department of Endocrinology of 2nd Affiliated Hospital of Dalian Medical University from February 2009 to February 2011. T2DM was diagnosed according to the 1999 World Health Organization criteria. Type 1 diabetes mellitus, hypertension, history of ischemic heart disease, renal impairment (serum creatinine >150 umol/L), and valvular heart diseases were excluded. The clinical conditions that could cause transient elevations in urinary albumin excretion, such as exercise, urinary tract infection, and febrile illness, were also excluded. According to the level of urinary albumin excretion (UAE), 210 patients of T2DM were divided into two groups: T2DM without microalbuminuria (T2DM1 group, UAE < 30 mg/24 h; 120 cases, 65 males and 55 females; mean age, 55.33 ± 9.58 years; age range, 27–78 years; diabetes duration, 6 months–5 years; treatment with diet or oral drugs) and T2DM with microalbuminuria (T2DM2 group, 30 mg/24 h < UAE < 300 mg/24 h; 90 cases, 48 males and 42 females; mean age, 58.89 ± 12.20 years; age range, 41–80 years; diabetes duration, 6 months–10 years; treatment with diet or oral drugs). The diagnosis of microalbuminuria was performed on the basis of one evaluation of UAE. The Ethics Committee of 2nd Affiliated Hospital of Dalian Medical University approved the study. All patients gave their informed consent to participate in the study.

### 2.2. Carotid Artery US Elastography

Carotid artery US elastography was performed using a real-time ultrasound scanner: Hitachi EUB 7500 with a linear 3–15 MHz probe (Hitachi Medical Systems, Tokyo, Japan). The scanner is equipped with a dedicated software to provide an accurate measurement of tissue elasticity. B-mode sonographic examinations were performed in transverse and longitudinal planes of the right carotid with the patients supine.

 In B-mode display, a midsection of straight carotid in longitudinal plane was chosen. The elastographic mode was performed to show double images of B-mode and elastography in the meantime. A rectangular region of interest (ROI) box covered at least 3 times of diameter of carotid artery. The probe was placed on the neck with proper pressure. The probe compression on the carotid region was standardized by real-time measurement displayed on a numerical scale (levels from 1–5). The levels from 2–4 were chosen in our study. It is important that the level of pressure is maintained constant throughout the examination. The ultrasound elastographic images were displayed with different color mapping including red (softest component), green (intermediate stiffness), and blue (hardest component) according to the different levels of strain. On a representative static image, the relative strain ratio (SR, blood to carotid arterial wall strain ratio) was measured. The first ROI (A) for the arterial wall strain was manually drawn and placed in approximate midpoint of posterior wall of displayed carotid artery. The second ROI (B) for the blood strain was placed in the center of arterial cavity. The distance between ROI (A) and ROI (B) should be almost the same in different scannings. SR was calculated by dividing strain value of the blood by that of carotid arterial wall. And SR was calculated automatically by an embedded software in the ultrasound scanner.

 All examinations were performed by two operators. The measurement was performed three times to obtain the mean values. The mean intraobserver and interobserver coefficients of variation were 4.5% and 3.1% for mean SR, respectively. The between-observer Pearson's correlation coefficient was 0.90 for mean SR.

### 2.3. Laboratory Assays

All laboratory assays were performed at the Clinical Laboratory Unit of the Second Affiliated Hospital of Dalian Medical University. The following laboratory parameters were obtained: total cholesterol (TC), triglyceride (TG), low density lipoprotein (LDL), high density lipoprotein (HDL), hemoglobin A1c (HbA1c), fasting plasma glucose (FPG), postprandial 2 hours plasma sugar (2hPG), and urinary albumin excretion (UAE). Serum concentrations of TC, TG, LDL, HDL, FBG, and 2hPG were measured by enzymatic method. HbA1C was measured by high performance liquid chromatography (BRO-RAD Company, USA). UAE was obtained by a 24-hour urine collection (by one determination). Body mass index (BMI) was calculated as weight in kilograms divided by height in meter squared. The intra- and interassay coefficients of variation of all techniques were less than 5%. Blood pressure was measured with a standard mercury sphygmomanometer after at least 10 min rest in the sitting position.

### 2.4. Statistical Analysis

The software of SPSS version 13.0 for Windows (SPSS Inc, IL, USA) was used for statistical analysis. Statistical significance between two groups was determined by Student's *t*-test. Continuous variables were expressed as $\bar{X}\pm \text{SD}$. Pearson correlation was used for correlation analysis. All tests were performed with *P* < 0.05 considered statistically significant.

## 3. Results

**Figure 1:**
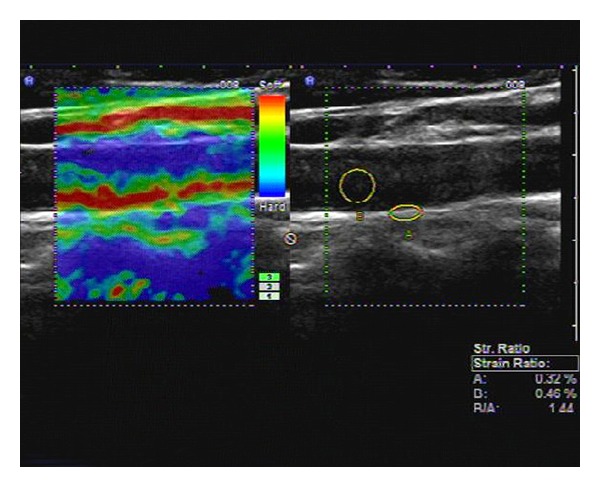
Longitudinal carotid artery image obtained in T2DM1 group on real-time US elastography. The left image shows the elastographic image in different colors representing different levels of strain. The right image shows the positions of ROI (A) and ROI (B), and the strain ratio (blood to carotid arterial wall strain ratio) as 1.44, calculated as the blood strain (B, 0.46%) divided by the arterial wall strain (A, 0.32%).

**Figure 2:**
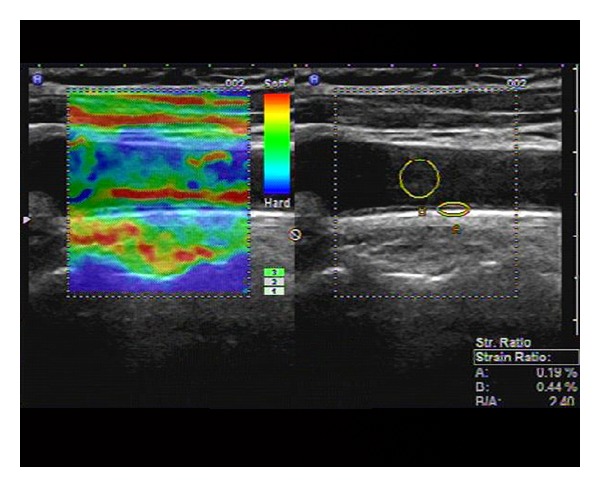
Longitudinal carotid artery image obtained in T2DM2 group on real-time US elastography. The left image shows the elastographic image in different colors representing different levels of strain. The right image shows the positions of ROI (A) and ROI (B), and the strain ratio (blood to carotid arterial wall strain ratio) as 2.40, calculated as the blood strain (B, 0.44%) divided by the arterial wall strain (A, 0.19%).

During the 24-month study period, 210 consecutive T2DM patients with or without microalbuminuria were screened. The characteristics of the 210 enrolled patients are shown in Table 1. The mean SR value ± SD of T2DM2 group (1.56 ± 0.45) was significantly higher than that of T2DM1 group (1.10 ± 0.24, *P* < 0.05) (Figures 1 and 2, Table 1). The mean values ± SD of 2hPG, BMI, TC, UAE, and HbA1c of T2DM2 group were significantly higher than those of T2DM1 group (*P* < 0.05 for all) (Table 1). There were no significant difference in systolic blood pressure, diastolic blood pressure, TG, LDL, HDL, FPG between T2DM1 and T2DM2 groups (*P* > 0.05 for all) (Table 1). In univariate analysis, SR was positively and significantly associated with UAE (*r* = 0.456, *P* < 0.05) and HbA1c (*r* = 0.435, *P* < 0.05), systolic blood pressure (*r* = 0.235, *P* < 0.05).

## 4. Discussion

The real-time US elastography is a newly developed dynamic technique which can obtain information on tissue stiffness and strain noninvasively [10, 11]. It can evaluate the degree of distortion of a tissue under an external force on the basis that the softer tissues deform easier than the harder one. In term of strain, strain represents the degree of deformation. Therefore, the soft tissue shows larger strain than the stiff tissue. In real-time US elastography, the strain images are showed in a color map. The color scale includes red, green, and blue colors. The red represents greatest elastic strain which is the softest, and the blue represents no strain which is the hardest. The green represents the mid level of strain. In our study, we used the strain ratio to reflect carotid arterial wall elasticity. The larger the SR is, the poorer the elasticity is.

 The present study has clearly shown that the mean SR value ± SD of T2DM patients with microalbuminuria was significantly higher than that in T2DM patients with normoalbuminuria. And SR was positively and significantly associated with UAE. The results suggested that microalbuminuria in T2DM may have an effect on elasticity of carotid arterial wall. Maybe there is a close relationship between atherosclerosis and diabetic nephropathy. Our findings were in accordance with the previous studies which evaluated the arterial wall elasticity in T2DM patients with microalbuminuria by pulse wave velocity (PWV) and echo-tracking technique [6–8]. But the mechanism underlying the relationship between microalbuminuria and atherosclerosis in T2DM patients is still unknown; in particular, little is known on the direct relationship between microalbuminuria and carotid arterial wall properties. There were reports that increased UAER could reflect a generalized vascular dysfunction which was caused by structural alterations, such as a reduction in the density of heparan sulfate-proteoglycan (HS-PG) and/or the sulphation of HS within the extracellular matrix of the glomerular basement membrane and vascular wall [12, 13]. HS-PG is synthesized in endothelial and myomedial cells. It is a normal component of glomerular basement membrane, endothelial vascular surface, and basement membrane of vascular smooth muscle cells. Furthermore, many proteins, such as lipoprotein lipase, tissue factor pathway inhibitor, platelet factor 4, and antithrombin III, are anchored to the vascular wall through interaction with the chains of HS-PG, which may enhance albuminuria and processes involved in atherogenesis [14–16]. Stehouwer et al. [17] found that microalbuminuria was linearly associated with impaired endothelium-dependent, flow-mediated vasodilation in elderly individuals without and with diabetes. It is possible that endothelial leakiness, as reflected by UAE, is in part a primary and possibly genetically determined vascular risk factor or that it mirrors the endothelial dysfunction featuring the atherosclerotic process or arises from the action of yet unknown risk factors [18].

In the present study, the result showed that the value of HbA1c in T2DM patients with microalbuminuria was significantly higher than that in T2DM patients with normoalbuminuria. And SR was positively and significantly associated with HbA1c. HbA1c maybe play an important role in the relationship between carotid arterial wall elasticity and microalbuminuria. HbA1c can accurately reflect longer-term glycemia. Clinically, HbA1c is regarded as a useful method of screening and diagnosing diabetes. And HbA1c has been accepted as the best marker for diabetic microvascular complications [19]. Moreover, HbA1c is associated closely with advanced glycation end products (AGEs) [20]. The previous study showed that AGEs are widespread in the diabetic vascular system and contribute to the development of atherosclerosis [21]. AGEs contribute to many microvascular and macrovascular complications through the formation of bridgings between molecules in the basement membrane of the extracellular matrix by joining the receptor for advanced glycation end products (RAGE). Concerning microalbuminuria, it was reported that the accumulation of AGEs in the glomerular and tubulointerstitial spaces correlates with the severity of diabetic nephropathy [22].

 Our study has its limitations. First, some T2DM patients have already been treated for diabetes and hyperlipidemia which may lead to inaccuracy of the results. Second, in the process of real-time US elastography, in order to get a relevant conclusion, either high or overly low pressure on the skin must be avoided. We chose the pressure of moderate degree according to a visual indicator displayed by a numerical scale on the screen. Nevertheless, the error induced by pressure cannot be avoided completely.

## 5. Conclusion

Our data show that there is an association between microalbuminuria and carotid arterial wall elasticity in T2DM patients. The decreased arterial wall elasticity caused by atherosclerosis may be related to the progression of diabetic nephropathy in T2DM patients. Real-time US elastography can evaluate carotid arterial wall elasticity in T2DM with microalbuminuria accurately and efficiently. However, larger and further studies are needed to confirm our results and hypothesis.

## Figures and Tables

**Table 1:** Characteristics of two groups.

Characteristic	T2DM1 group (*n* = 120)	T2DM2 group (*n* = 90)	*P *value
Systolic blood pressure (mmHg)	129.8 ± 15.5	130 ± 16.5	0.780
Diastolic blood pressure (mmHg)	81.8 ± 9.3	80.7 ± 9.5	0.830
2hPG mmol/L	9.42 ± 4.05	12.93 ± 4.80	0.008
FPG mmol/L	9.43 ± 3.66	9.35 ± 4.07	0.940
BMI kg/m^2^	25.66 ± 4.08	27.80 ± 3.22	0.029
TC mmol/L	4.88 ± 1.17	5.51 ± 0.87	0.024
TG mmol/L	1.68 ± 1.27	2.21 ± 1.36	0.125
LDL mmol/L	3.20 ± 1.20	3.30 ± 0.80	0.584
HDL mmol/L	0.99 ± 0.27	0.98 ± 0.24	0.897
HbA1c %	8.36 ± 2.33	9.45 ± 1.86	0.046
UAE mg/24 h	12.22 ± 8.57	96.55 ± 64.67	0.000
SR	1.10 ± 0.24	1.56 ± 0.45	0.000
